# Distribution of mastitis-causing pathogens isolated from subclinical mastitis cases in Wisconsin dairy herds with low bulk tank somatic cell counts

**DOI:** 10.3168/jdsc.2026-1100

**Published:** 2026-09-07

**Authors:** Maria Jose Fuenzalida, Rainna Simone, Carolina Pinzón-Sánchez, Ashley Hanneman, Heather Schlesser

**Affiliations:** 1 Animal and Food Science Department, University of Wisconsin–River Falls, River Falls, WI 54022; 2 Division of Extension, University of Wisconsin–Madison, Madison, WI 53716

## Abstract

The objective of this cross-sectional study was to characterize the distribution of mastitis-causing pathogens (MCP) in cases of subclinical mastitis (SM) across 14 Wisconsin dairy farms with a history of low bulk tank somatic cell counts (BTSCC). Herds were eligible for enrollment if their average BTSCC during the previous 12 mo was ≤150,000 cells/mL. A total of 455 cows were enrolled based on recent DHI(A) records indicating SCC ≥200,000 cells/mL. Milk samples were collected from functional quarters (n = 1,811), and SCC was measured in 1,756 samples due to insufficient milk volume or sample loss. Only quarter milk samples with SCC ≥200,000 cells/mL were used for microbiological culture. The quarter-level prevalence of SM was 33.3% (585/1,756). Among SM cases, 22.6% (132/585) were contaminated, 28.4% (166/585) were negative for microbial growth (NG), and 49.0% (287/585) had an identifiable microbe. After excluding contaminated samples, NG and non-*aureus* staphylococci and mammaliicocci (NASM; n = 120) were the most frequent diagnoses. *Enterococcus* spp. (ECS, 8.8%) and *Lactococcus* spp. (LAC, 6.8%) were identified more frequently than environmental streptococci (6.6%). Among NASM, *Staphylococcus chromogenes* (13.9%) was the most frequently identified species. This study did not have enough power to identify significant variability for MCP distribution between farms. These findings highlight that, although NG and NASM remain the predominant microbiological culture results, ECS and LAC are distinct and important contributors to SM that may be underestimated by traditional phenotypic methods. Overall, these findings emphasize the importance of pathogen-level surveillance for developing targeted mastitis control strategies in high-performing dairy systems.

## Graphical Abstract

**Figure:**
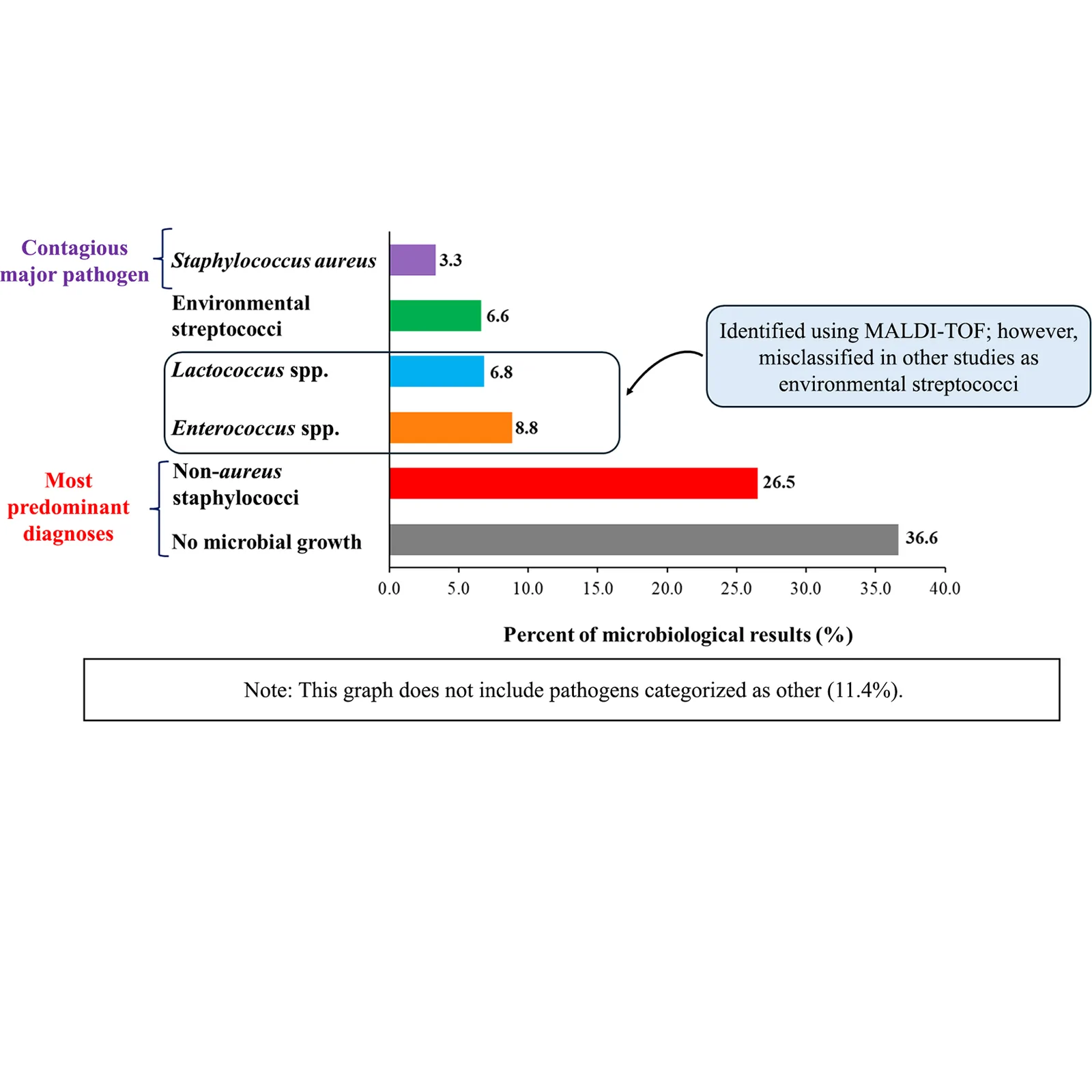

Mastitis remains one of the most economically important diseases affecting dairy cattle worldwide (Liang et al., 2017; NAHMS, 2025). Most cases of mastitis are caused by bacteria that can induce either subclinical or clinical states of the disease, with cows potentially transitioning between states depending on the causative pathogen. Subclinical mastitis (**SM**) is characterized by elevated SCC in the absence of visible clinical signs and is commonly monitored at the herd level using bulk tank somatic cell count (**BTSCC**), an established indicator of milk quality and udder health (Rowbotham and Ruegg, 2015). Historically, elevated BTSCC has been associated with the presence of major contagious pathogens, particularly *Staphylococcus aureus* and *Streptococcus agalactiae*, and substantial progress in mastitis control programs has resulted in a marked reduction in the prevalence of these organisms in many dairy regions (Makovec and Ruegg, 2003; Keefe, 2012; Ruegg, 2017). Nowadays, BTSCC averages 170,000 cells/mL in the United States (NAHMS, 2025), and the relative importance of environmental pathogens such as *Escherichia coli*, non-*aureus* staphylococci and mammaliicocci (**NASM**), and environmental streptococci (**ENV**) has increased, reflecting shifts in pathogen ecology and management practices (Bradley, 2002; Makovec and Ruegg, 2003; Oliveira et al., 2013).

The etiology of SM cases varies considerably across studies and geographic regions. A substantial proportion of cases are microbiologically negative (**NG**), with reported rates ranging from 26.4% to 67.2%, followed by NASM and other gram-positive environmental pathogens (Persson et al., 2011; Bortolami et al., 2015; Rowbotham and Ruegg, 2016; Bobbo et al., 2017; Condas et al., 2017; Zalewska et al., 2025). Among NASM, the most frequently isolated and prevalent species are *Staphylococcus chromogenes*, with wide variation in prevalence for the other NASM: *Staphylococcus simulans*, *Staphylococcus xylosus*, *Staphylococcus epidermidis*, and *Staphylococcus hyicus* (Gillespie et al., 2009; Condas et al., 2017). Reports from Poland indicate that *Mammaliicoccus sciuri* predominates, followed by *Mammaliicoccus vitulinus*, *Staphylococcus xylosus*, and *Staphylococcus chromogenes* (Zalewska et al., 2025). In contrast, other environmental pathogens, such as enterococci and lactococci, are less consistently described in the literature (Persson et al., 2011; Bobbo et al., 2017), and limited data are available to estimate their prevalence at the cow and herd levels in cases of SM. This variability may reflect differences in study design, culture and diagnostic methodologies, and herd-level management factors. To our knowledge, no published studies have characterized the distribution of pathogens associated with SM in relation to herd-level BTSCC. With most US dairy herds now maintaining relatively low BTSCC through established mastitis control practices (Ruegg, 2017; NAHMS, 2025), identifying the pathogens responsible for SM in these high-performing herds is important for refining prevention and control strategies. The objective of this study was to characterize mastitis-causing pathogens (**MCP**) isolated from cows with SM in dairy herds in Wisconsin with low BTSCC.

A total of 12 commercial and 2 research dairy farms were enrolled in a cross-sectional study between August 2024 and August 2025. Dairy cows were milked in parallel (farms B–L) or rotary (farms A, M, and N) parlors. All cows were milked 3 times per day except for cows in farm K. Eligibility criteria for farms included participation in monthly DHI(A) testing that included individual cow SCC data, average BTSCC from the last 12 mo ≤150,000 cells/mL, use of computerized herd records, and willingness to participate in this project. Only cows experiencing SM, as determined by DHI(A) test data, were eligible for enrollment. Subclinical mastitis was defined as inflammation of the mammary gland in the absence of visible clinical signs, as indicated by quarter SCC (**QSCC**) ≥200,000 cells/mL (Ruegg and Erskine, 2014). All eligible cows had SCC ≥200,000 cells/mL on 1 or both of the 2 most recent monthly DHI(A) tests, with the most recent test conducted within 14 d before enrollment. Cows were excluded if they received any antimicrobial treatment within 30 d of enrollment or experienced clinical mastitis (**CM**) on the day of sample collection.

Study personnel conducted 1 farm visit per dairy to collect quarter milk samples from cows experiencing SM. A convenience sample of 20 to 45 cows experiencing SM were sampled per farm. The final number of samples collected reflected a balance between the prevalence of SM in the herd and the practical feasibility dictated by available resources and personnel. Cows were sampled on a first-come, first-served basis. Due to time constraints and to minimize disruption of the milking routine, only 2 milk samples per quarter were collected. One sample was aseptically collected for milk microbiological culture (**MMC**), and the other sample was preserved with bronopol and transported to a commercial DHI(A) laboratory (AgSource Laboratory, Menomonie, WI). Only quarter milk samples with ≥200,000 cells/mL were used for MMC. Frozen quarter milk samples were thawed at room temperature and plated at the University of Wisconsin–River Falls laboratory (River Falls, WI) following National Mastitis Council guidelines (NMC, 2017). We inoculated 100 μL of milk onto half of a blood agar plate (Trypticase soy agar with 5% sheep blood; BD Diagnostic Systems, Becton Dickinson, Franklin Lakes, NJ). A 100-µL inoculum was used following previously described methods (Fuenzalida et al., 2015; Fuenzalida and Ruegg, 2019a,b) to increase the probability of recovering microbial species present at low concentrations. After inoculation, plates were incubated at 37°C for 24 to 48 h. The MMC result was defined at the quarter level. In this study, IMI was defined as the isolation of 100 cfu/mL of identical colonies. However, for *Staphylococcus aureus*, IMI was defined as the isolation of at least 10 cfu/mL. Mixed infection was defined as at least 10 colonies of 2 types of bacteria found in the same milk sample. Milk samples were considered contaminated when 3 or more colony types were isolated from the same sample. Nonsignificant growth was defined as growth of less than 100 cfu/mL of 1 or 2 types of bacteria from a single milk sample. The presence of *Mycoplasma* spp. was not evaluated in this study. The NG MMC results could indicate either the absence of an IMI or failure to recover microorganisms present in the mammary gland (Lago, 2009; Metzger, 2018).

Pure colonies were frozen and stored at −80°C until further analysis. Isolates were subsequently cultured onto blood agar and sent to the Wisconsin Veterinary Diagnostic Laboratory (Madison, WI) for speciation using MALDI-TOF MS with the Bruker Daltonics reference database. For analysis, the results of quarter milk samples with nonsignificant growth were classified as NG. Pathogens identified to the species level were categorized into MCP using the following MMC classification system: NASM; *Enterococcus* spp. (**ECS**); *Lactococcus* spp. (**LAC**); ENV; *Staphylococcus aureus* (**AUR**); organisms with low prevalence or unresolved identification were grouped into the “other” category, which included yeast, fungi, gram-negative organisms, gram-positive rods (not resolved to species by MALDI), and mixed infections. Thus, MCP categories included NG, NASM, ECS, LAC, ENV, AUR, and other.

Statistical analyses were performed using SAS (version 9.4; SAS Institute, 2023). Quarter was considered the observational unit. Categorical outcomes were MCP and contaminated milk samples (yes or no). Continuous outcomes were QSCC at enrollment. Categorical explanatory variables were parity group (1 and 2, and ≥3), CM history (yes or no), quarters (left front, **LF**; right front, **RF**; left rear, **LR**; right rear, **RR**), and 4 functional quarters (yes or no). Continuous variables included milk yield (kg/cow per day), previous SCC from 1 to 21 d before enrollment, and DIM. Somatic cell count data were log-transformed [log_10_SCC = log_10_(SCC × 1,000)] to follow a normal distribution.

Descriptive statistics of farm characteristics, QSCC, and MCP were performed using PROC FREQ in SAS. Binomial proportions for MCP (presence or absence of each pathogen) were estimated using PROC FREQ. This method provided both asymptotic (Wald) and exact (Clopper–Pearson) 95% CI, ensuring robust estimation, particularly for small proportions. Univariate associations between categorical outcomes and selected risk factors were evaluated using a chi-squared test (PROC FREQ), and differences in continuous variables were assessed using ANOVA (PROC GLM). The association between MCP category (NG, NASM, ECS, LAC, ENV, AUR, and other) and QSCC was evaluated using a univariable linear mixed-effects model (PROC MIXED), with MCP category included as a fixed effect and farm included as a random effect to account for clustering of observations within farms. The LSM and 95% CI for each MCP category were estimated using the LSMEANS statement, and pairwise comparisons among MCP categories were performed using the Tukey–Kramer adjustment for multiple comparisons. Statistical significance was defined as *P* < 0.05.

A posteriori power analysis indicated that ∼650 quarter-level MMC results were required to achieve 80% power at a significance level of 0.05. This calculation accounted for the clustering of observations within cows and was based on the number of quarter-level SM cases with complete MMC results data, excluding contaminated samples and the expected proportions of MCP categories.

Mixed-effects logistic regression models were fit using PROC MIXED. Selected risk factors associated with milk sample contamination status (contaminated vs. noncontaminated) and MCP were included in the models. For model convergence, MCP categories were re-categorized as (1) NG and NASM, and (2) other (ECS, LAC, ENV, and AUR). Goodness of fit was assessed using Akaike's information criterion, Bayesian information criterion, and likelihood ratio tests. For model 1, all SM cases with MMC data were included in an intercept-only (null) model with a random intercept for farm to assess between-farm variation in contamination status. The null hypothesis was that there is no variability between farms in the probability of milk sample contamination. Subsequently, models including within-farm explanatory variables (e.g., cow- and quarter-level factors) were compared with the intercept-only model. For model 2, a subset of the data set excluding contaminated samples was used to assess the differences in MCP categories (NG and NASM vs. other) across farms. Farm was included as a fixed effect, and cow nested within farm was included as a random effect to account for clustering of observations within cows. The null hypothesis was that there is no between-farm variability in MCP category classification among enrolled herds. A priori power analysis was conducted using G*Power (v. 3.1.9.7; Franz Faul, Universität Kiel, Germany) based on a chi-squared approximation, and the required sample size was adjusted for clustering using a design effect. Models were chosen based on parsimony and goodness of fit.

Enrolled herds ranged in size from 109 to 3,888 lactating cows. The average milk yield was 42.4 kg/cow per day, ranging from 35.5 to 45.4 kg/cow per day. The geometric mean BTSCC during the last 12 mo before enrollment was 113,691 cells/mL (range: 81,468–149,231 cells/mL, Table 1). Both milk yield and BTSCC varied between farms (*P* < 0.001).

**Table 1:** Descriptive characteristics of the 14 Wisconsin dairy herds enrolled

Farm	BTSCC,^1^ cells/mL	Milk yield,^2^ kg	Lactating cows per herd, n	Cows enrolled,^3^ n	Quarters enrolled,^4^ n	Quarters tested for SCC,^5^ n	Quarters with SM,^6^ n	Quarters analyzed,^7^ n
A	106,841^a^	45.4^a^	1,370	20	80	77	17	16
B	93,872^c^	42.8^b^	1,451	26	104	101	35	29
C	81,468^d^	43.6^a^	2,458	20	78	61	20	17
D	134,343^b^	42.8^b^	1,189	41	162	157	56	55
E	119,417^b^	42.0^b^	478	28	112	111	46	29
F	117,572^b^	44.3^a^	1,128	40	160	158	53	48
G	116,697^b^	44.3^a^	816	35	140	138	50	46
H	105,789^a^	42.8^b^	679	40	160	156	51	45
I	97,839^a^	35.4^d^	1,199	24	96	91	24	15
J	130,288^b^	42.1^b^	2,006	45	180	179	77	46
K	149,231^b^	41.9^b^	109	22	88	87	16	13
L	98,376^a^	43.7^a^	788	29	116	111	28	23
M	124,336^b^	39.5^c^	3,888	40	157	155	57	33
N	133,532^b^	38.7^c^	3,656	45	178	174	55	38

a–d Within a column among farms, means (ANOVA) with different superscripts differ (*P* < 0.05).

1 Bulk tank somatic cell count (BTSCC) from DHI(A) testing for the last 12 mo before enrollment. Bulk tank SCC values were log_10_-transformed, then back-transformed to SCC per milliliter.

2 Milk yield per cow per day from DHI(A) testing for the last 12 mo before enrollment.

3 Cows selected and used for quarter milk sample collection. These cows met the enrollment criteria: cows had ≥200,000 cell/mL on either or both most recent monthly SCC tests before enrollment and did not receive any antimicrobial treatment within 30 d before enrollment in the study, or experienced clinical mastitis on the day of sample collection.

4 Number of quarters used for milk sample collection.

5 Includes only quarters with complete SCC records; missing values were due to insufficient milk volume or sample loss.

6 Quarter milk samples with SCC ≥200,000 cells/mL (n = 585).

7 Excluding contaminated samples (n = 453).

A total of 455 lactating cows were enrolled in the study (ranging from 20 to 45 cows per herd, Table 1). Among the enrolled cows, 11% (53/455) had only 3 functional quarters, ranging from 1 to 13 cows per herd. Overall, 1,811 functional quarters were included in the study, corresponding to 78 to 180 quarters enrolled per farm (Table 1). Milk samples were collected from functional quarters, and SCC measurements were available for 97% of quarters (n = 1,756, 78.2%–99.4%, Table 1), with missing data primarily due to insufficient milk volume or sample loss by study or laboratory personnel.

Based on the study definition of SM, 33.3% (585/1,756) of the tested quarters were classified as experiencing SM. The proportion of affected quarters varied among farms, ranging from 18.4% (16/87; farm K) to 43.0% (77/179; farm J; χ^2^ = 31.3, *P* = 0.003). The LSM of quarter log_10_SCC was associated with SM status (*P* < 0.001) but not with quarter affected (*P* = 0.866). Among quarters experiencing SM, log_10_SCC (mean ± SE) was 6.02 ± 0.04 (LF), 6.03 ± 0.04 (RF), 6.06 ± 0.03 (LR), and 6.03 ± 0.03 (RR). Among healthy quarters (n = 1,172), log_10_SCC was 4.46 ± 0.02 (LF), 4.44 ± 0.02 (RF), 4.43 ± 0.03 (LR), and 4.42 ± 0.02 (RR). Among quarters with SM, the proportion with complete MMC results ranged from 59.7% (46/77; farm J) to 94.1% (16/17; farm A). The primary reason for exclusion was sample contamination, and this proportion varied among farms (χ^2^ = 54.2, *P* < 0.001). Contamination status varied among farms. The null model including only a random intercept for farm demonstrated better goodness of fit than models adjusting for within-farm factors (*P* > 0.305). These findings indicate that most of the observed variability in contamination status was attributable to farm-level clustering rather than to within-farm characteristics.

Among quarters classified as having SM, the distribution of MCP categories was as follows (mean ± SE; asymptotic and exact 95% CI): NG was 36.6% ± 2.3% (32.2–41.3); NASM was 26.5% ± 2.1% (22.4–30.8); ECS was 8.8% ± 1.3% (6.2–11.8); LAC was 6.8% ± 1.2% (4.5–9.6); ENV was 6.6% ± 1.2% (4.3–9.3); AUR was 3.3% ± 0.8% (1.7–5.4%); and other was 11.3% ± 1.5% (8.3%–14.5%). Considerable variability in MCP distribution was observed among farms (Figure 1); however, after accounting for within-cow clustering and multiple comparisons, no significant pairwise differences were detected (*P* = 0.07). Based on the observed data, the effect size for differences in pathogen distribution among farms was moderate (Cohen's w = 0.26). The present study was underpowered to detect statistically significant differences among farms.

**Figure 1:**
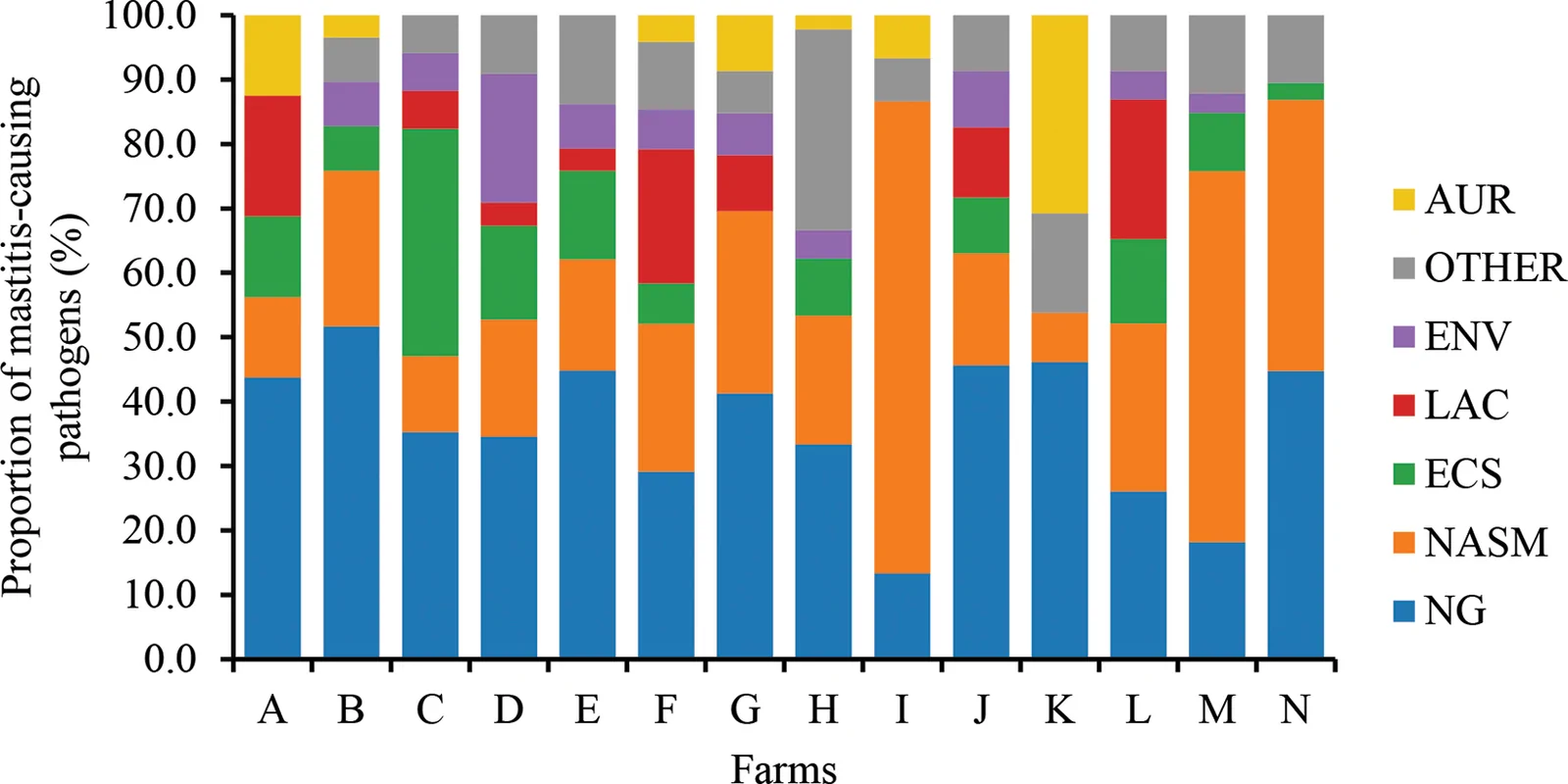
Farm-level proportional distribution of mastitis-causing pathogen categories in quarter milk samples from subclinical mastitis cases (n = 453) across study herds. NG = no microbial growth (n = 166); NASM = non-*aureus* staphylococci and mammaliicocci (n = 120); ECS = *Enterococcus* spp*.* (n = 40); LAC = *Lactococcus* spp. (n = 31); ENV = environmental streptococci (n = 30); other = other pathogens (n = 51); AUR = *Staphylococcus aureus* (n = 15). Species-level identification was conducted for all groups except NG, with results aggregated for presentation.

The LSM for QSCC varied among MCP categories (*P* < 0.0001). The QSCC LSM (mean ± SEM; 95% CI) were 5.95 ± 0.04 (5.87–6.03) for NG, 5.91 ± 0.04 (5.82–6.00) for NASM, 6.17 ± 0.08 (6.02–6.32) for ECS, 6.22 ± 0.09 (6.05–6.39) for LAC, 6.56 ± 0.09 (6.39–6.74) for ENV, 6.04 ± 0.12 (5.80–6.29) for AUR, and 6.16 ± 0.07 (6.03–6.30) for other. Pairwise comparisons showed that QSCC LSM was greater for ENV than for AUR, ECS, NASM, NG, and other (*P* < 0.05). In addition, QSCC LSM was greater for LAC than for NASM, greater for ECS than for NASM, and greater for other than for NASM (*P* < 0.05).

Of the MMC results, 22.6% (132/585) were classified as contaminated. After excluding contaminated samples, 36.6% (166/453) of the remaining samples yielded no microbial growth, and 63.4% (287/453) yielded identifiable microorganisms (Table 2). Among samples with microbial growth (Table 2), gram-positive bacteria predominated, particularly NASM, with *Staphylococcus chromogenes* (13.9%) and *Staphylococcus simulans* (4.9%) being the most frequently identified species. Other common gram-positive isolates included *Enterococcus saccharolyticus* (7.7%), *Lactococcus lactis* (3.5%), and members of the *Streptococcus bovis/Streptococcus equinus* complex (4.4%). *Staphylococcus aureus* accounted for 3.3% of samples. Gram-negative bacteria were infrequently detected (1.6%). Fungi and yeast species represented a small proportion of isolates, with *Pichia kudriavzevii* (2.9%) being the most common. *Prototheca* spp*.* (1.3%) and mixed infections (1.8%) were also identified.

**Table 2:** Milk microbiological culture results in cases of subclinical mastitis (n = 585)

Pathogen	n	%^1^
Gram-negative bacteria^2^	7	1.55
Gram-positive bacteria (GP)		
* Staphylococcus chromogenes*	63	13.91
* Enterococcus saccharolyticus*	35	7.73
* Staphylococcus simulans*	22	4.86
* Streptococcus bovis/Streptococcus equinus* complex	20	4.42
* Lactococcus lactis*	16	3.53
* Staphylococcus aureus*	15	3.31
* Staphylococcus epidermidis*	13	2.87
* Lactococcus species*	8	1.77
* Staphylococcus hyicus/agnetis*	8	1.77
* Lactococcus garvieae*	7	1.55
* Staphylococcus haemolyticus*	6	1.32
* Streptococcus dysgalactiae*	5	1.10
* Streptococcus uberis*	5	1.10
* Staphylococcus* species	4	0.88
* Staphylococcus borealis*	2	0.44
* Enterococcus cecorum*	1	0.22
* Enterococcus faecium*	1	0.22
* Enterococcus thailandicus*	1	0.22
* Enterococcus* species	1	0.22
* Staphylococcus gallinarum*	1	0.22
* Staphylococcus microti*	1	0.22
* Streptococcus* species	1	0.22
Fungi		
* Pichia kudriavzevii*	13	2.87
Diutina rugosa	5	1.10
Yeast, nonspeciated	2	0.44
Cyberlindnera fabianii	1	0.22
* Magnusiomyces capitatus*	1	0.22
Other^3^	22	4.86
No growth	166	36.64

1 Proportions were calculated without including the contaminated samples (n = 132).

2 Gram-negative bacteria include *Escherichia coli, Klebsiella pneumoniae*, gram-negative cocci, *Pasteurella multocida, Pseudomonas aeruginosa*, and *Serratia marcescens.*

3 Other includes GP pleomorphic, GP rod, gram-positive cocci, *Bacillus* spp., *Corynebacterium xerosis, Prototheca* spp., and mixed infection.

The cross-sectional study included cows from 14 Wisconsin dairy herds that voluntarily enrolled in the field trial. These herds exhibited exceptionally high milk quality and production, as evidenced by lower BTSCC and greater milk yield per cow per day compared with state and national averages (NAHMS, 2025). Herd size was similar to previously reported state averages for high-producing dairy farms (NASS, 2022). Findings of this study can be extrapolated to dairy farms with similar characteristics.

This study faced 4 limitations. One was the relatively high proportion of contaminated samples. The use of a greater inoculum volume may have contributed to the observed contamination rate, compared with standard methods using smaller inoculum volumes (NMC, 2017). To adjust for the exclusion of contaminated samples, herd was included in the statistical analyses to account for farm-level variation in contamination rates, thereby reducing the potential influence of differences in contamination on estimates of microorganism distribution. A second limitation was that we aimed to enroll a specific number of cows per farm based on a sample size calculation that included the expected distribution of MCP and anticipated exclusion of ∼10% of milk samples due to contamination or poor collection. The enrollment of a specific number of cows per farm was not consistently achieved due to variability in SM prevalence across dairy herds and logistical limitations, as sampling needed to be conducted without substantially disrupting routine milking procedures. A third limitation was the potential for selection bias because eligible cows were not randomly selected for quarter milk sample collection. Nevertheless, among sampled cows, SCC data were obtained for the majority of the quarters, allowing a reliable distinction between quarters experiencing SM and healthy quarters. As highlighted by Levin (2006), the external validity of cross-sectional studies depends on the sampling frame and on how observational units are defined and represented, which in this study corresponded to discerning between SM and healthy quarters, then determining the etiology of pathogens causing SM cases. A fourth limitation was associated with MALDI as a method for identification, as some isolates may not have been reliably identified to the species level (Singhal et al., 2015). Identification was performed using 1 of the 2 major commercial MALDI reference databases, which provides reliable identification for most clinically relevant microorganisms. Although these limitations may affect generalizability, the findings remain applicable to dairy herds managed under similar field conditions.

Our findings indicated that the quarter-level prevalence of SM among eligible cows was 33.3%. This estimate is greater than the cow-level prevalence of SM reported in previous studies conducted in dairy herds with similar characteristics (Rodrigues et al., 2005; Fuenzalida et al., 2015). However, such differences are expected, as quarter-level prevalence is typically greater than cow-level prevalence due to the larger number of units at risk when quarters, rather than cows, are used as the denominator (10.7% at the cow level and 12.7% at the quarter level; McDougall et al., 2025). In contrast, our average quarter-level prevalence of SM among eligible cows was lower than the cow-level prevalence of 46.4% reported in a retrospective observational study conducted in Brazil (Busanello et al., 2017) and the estimated cow-level prevalence of 46.1% in North America (Rasmussen et al., 2024). These differences should be interpreted cautiously, as the cited studies differed in study design, case definitions, and inclusion criteria. Our study estimated quarter-level prevalence among eligible cows with elevated SCC, thereby enriching the study population of SM cases.

Quarter SCC varied among MCP categories. In the present study, quarters classified as NG and NASM had the lowest estimated QSCC, whereas ENV had the highest, with LAC, ECS, and other also exhibiting numerically higher QSCC. This pattern is consistent with previous studies reporting greater SCC associated with ENV and other major mastitis pathogens than with NASM. Malek dos Reis et al. (2011) reported greater QSCC for *Streptococcus dysgalactiae* (log_10_SCC = 5.98), *Staphylococcus aureus* (5.94), and *Streptococcus uberis* (5.52) compared with culture-negative quarters (4.93) and NASM (5.34). Similarly, Taponen et al. (2022) observed greater QSCC values for *Staphylococcus aureus* (5.72) and *Staphylococcus simulans* (5.51) than other NASM. Thorberg et al. (2009) reported lower SCC among cows infected with *Staphylococcus chromogenes*, *Staphylococcus epidermidis*, and *Staphylococcus simulans* (∼5.26–5.42). Direct comparisons should be interpreted cautiously, as previous studies differed in study design, IMI definitions, SCC inclusion criteria, and pathogen classification.

After NG, the most frequent MCP categories were NASM, ECS, and LAC. Other isolates, including ENV and AUR, occurred at lower frequencies. The NG and NASM are consistently reported as the 2 most common MMC results in SM cases, with ENV typically ranking third or fourth, depending on the presence of contagious pathogens within dairy herds (Barrett et al., 2005; Persson et al., 2011; Rowbotham and Ruegg, 2016; Bobbo et al., 2017; Condas et al., 2017). Although NG and NASM were also the 2 most prevalent MMC results, we observed a greater prevalence of ECS and LAC compared with ENV, which contrasts with previous reports (Barrett et al., 2005; Persson et al., 2011; Bobbo et al., 2017; Condas et al., 2017). A likely explanation for this difference is the methodology used to differentiate ECS and LAC from ENV. These bacteria are often grouped under ENV when using phenotypic and biochemical diagnostic methods (Werner et al., 2014), potentially leading to underestimation of the true proportions of ECS and LAC in earlier studies. To our knowledge, this is one of the first reports that describes ECS and LAC as distinct MMC results in dairy herds with low BTSCC, providing a more refined characterization of MCP distribution under these herd conditions. In conclusion, our results suggest potential differences in MCP distribution across the enrolled dairies. This variability between farms should be interpreted with caution; however, it provides preliminary evidence that pathogen profiles are not uniform, even among herds with similar BTSCC and management characteristics. Such heterogeneity may reflect differences in analytical sensitivity, environmental conditions, milking practices, cow-level risk factors, and pathogen transmission dynamics. Consequently, these findings highlight the importance of considering farm-specific MCP patterns when designing mastitis prevention and control programs. Relying on generalized assumptions about pathogen distribution may limit the effectiveness of interventions, whereas targeted strategies based on the predominant pathogens within each herd are more likely to improve udder health outcomes. Despite the need for larger sample sizes to confirm these observations, our data underscore the value of pathogen-level surveillance at the farm level as a key component of successful mastitis control in high-performing dairy systems.
